# Perceived Vulnerability and Severity Predict Adherence to COVID-19 Protection Measures: The Mediating Role of Instrumental Coping

**DOI:** 10.3389/fpsyg.2021.674032

**Published:** 2021-07-06

**Authors:** José Luis González-Castro, Silvia Ubillos-Landa, Alicia Puente-Martínez, Marcela Gracia-Leiva

**Affiliations:** ^1^Educational Science Department, University of Burgos, Burgos, Spain; ^2^Health Science Department, University of Burgos, Burgos, Spain; ^3^Social Psychology Department, University of the Basque Country, Gipuzkoa, Spain

**Keywords:** COVID-19, protection measures, vulnerability, severity, instrumental coping, self-efficacy, longitudinal study

## Abstract

The COVID-19 disease has caused thousands of deaths worldwide and required the rapid and drastic adoption of various protective measures as main resources in the fight to reduce the spread of the disease. In the present study we aimed to identify socio cognitive factors that may influence adherence to protective measures toward COVID-19 in a Spanish sample. This longitudinal study analyzes the predictive value of perceived severity and vulnerability of infection, self-efficacy, direct exposure to the virus, and instrumental focused coping style for adhering to infection protection behaviors during the first months of the COVID-19 pandemic. It also tests sex and age differences in these factors and changes over time. A two-wave longitudinal study (*N* = 757) was conducted in March and April 2020 starting the day after a strict national lockdown was decreed in Spain. A path analysis was used to test direct and indirect effects between vulnerability and the adherence to protective behaviors. Results suggest that individuals' perceived severity and vulnerability to COVID-19 and instrumental coping strategies are related to the use of more protective behaviors. This coping strategy mediates the effect of perceived vulnerability on engaging in protective behaviors, and this effect depends on direct exposure to COVID-19 and perceived self-efficacy moderators. Results suggest that recognizing one's own abilities to engage in instrumental actions may facilitate adherence to protective measures in people who had not been directly exposed to COVID-19. Therefore, adopting instrumental coping strategies to manage an individual's perceived vulnerability to infection may positively impact the adherence to protective behaviors, especially during the onset of an unexpected threat and when there is no prior direct experience with the situation.

## Introduction

On January 7th 2020, a novel coronavirus was identified by Chinese authorities and temporarily named 2019-nCoV. Due to its rapid worldwide spread, the World Health Organization (2020) declared COVID-19, as the disease was now termed (the virus is defined as SARS-CoV-2), a pandemic on March 11th 2020. As a consequence of the pandemic declaration, public health agencies throughout the world proposed several measures to contain or mitigate the virus transmission including one or various confinements, lockdowns, and multiple social distancing measures (Coroiu et al., 2020). During a pandemic, and until effective vaccines are rolled out to the whole population, the adherence to measures thought to protect from contagion are not only a way of reducing one's risk of developing an illness but also of spreading the infection among the population. Although protective measures are subject to constant scrutiny and have changed over time, from the onset of the pandemic there have been certain measures (social distancing, wearing facemasks, or using hand sanitizer) largely accepted as adequate for reducing the spread of the virus (Kennedy et al., 2020). Many of these measures are novel to most societies (especially Western ones) and result in relevant lifestyle changes for the general population. Moreover, complying with these measures implies accepting changes enforced by governments that may restrict individual and social rights. As such, they are measures that deeply affect our perception of social relationships and interaction patterns. Complying with these novel social norms is difficult. For instance, Smith et al. (2020) show that adherence to lockdown measures was poor in the United Kingdom during the first phase of the lockdown (May 2020). It is important to understand the barriers and facilitators that lead people to adhere, or not, to these measures. This requires that those involved in both drafting and maintaining these “new” social norms understand the psychological determinants of these behaviors (Makhanova and Shepherd, 2020).

### Theoretical Background

This study is based on socio-cognitive constructs derived from the Health Belief Model (HBM) (Rosenstock, 1974; Janz and Becker, 1984). As Raude et al. (2020b) mention, socio-cognitive factors seem to play a more important role than sociocultural and psychosocial factors in adopting COVID-19 related preventive health behaviors.

HBM is an expectancy-value theory drawing extensively on threat perception and the behavioral evaluation of a situation as a framework for predicting changes in health behaviors. This model states that an individual's protective behavior is influenced by their perceived severity, perceived vulnerability, perceived benefits and perceived barriers to engage in protective behaviors (Rosenstock, 1974). Severity refers to beliefs about how serious the consequences of the condition would be, while vulnerability addresses the extent to which an individual feels vulnerable to the situation (Champion and Skinner, 2008). Perceived benefits refer to the effectiveness and availability of taking a particular course of action, and perceived barriers are the negative aspects related to following the course of action (Rosenstock, 1974). In this study, we will analyze specifically the importance of threat perceptions that include two components: perceived severity and vulnerability. Individuals with different global and personal perceptions (severity and vulnerability) of COVID-19 could show different behavioral reactions toward COVID-19. Li et al. (2020), Yildirim and Güler (2020), or Hills and Eraso (2021) mention that, in general, perceived susceptibility and severity of the disease seem to increase engagement and compliance with preventive behaviors toward COVID-19.

Moreover, engaging in protective behaviors (such as adherence to recommended health prevention measures) not only depends on a person's appraisal of a threat and its severity but on the perceptions about one's ability to engage in preventive behaviors (Rogers, 1975). Rosenstock et al. (1988) stated, based on Social Cognitive Theory, that the perceived barriers component of the HBM should include feelings of confidence in one's perceived ability to perform a protective behavior. Maddux and Rogers (1983) found that self-efficacy was the most powerful predictor of behavioral intentions. Self-efficacy is defined (Bandura, 1997) as the belief a person has in their ability to cope with life difficulties and challenges, control their function and the events that affect their lives, assess situations accurately and seek appropriate ways of coping with difficulties and obstacles. In Shahnazi et al.'s (2020) study, participants who had high-perceived self-efficacy were more inclined toward adopting preventive behaviors toward COVID-19. Jørgensen et al. (2020) results also show that perceived efficacy predicted self-reported engagement in protective behaviors during the COVID-19 pandemic.

The widespread high perception of threat of contagion also leads to engaging in coping strategies to avoid contracting SARS-CoV-2. In fact, an important line of research recognizes the relevance of including coping theory to better understand the behaviors and responses to stress during the pandemic (Chen and Bonanno, 2020; Rana et al., 2021). Coping is defined as a person's cognitive and behavioral efforts to manage specific external and/or internal demands that are considered taxing and go beyond a person's resources (Lazarus, 1999). This current study analyzes problem-focused coping (active and planning strategies) whose purpose is to solve, or change, the situation in which there is a threat of contracting the virus. Dual-phase behavior models, such as the Health Action Process Approach (HAPA; Schwarzer, 2008; Schwarzer and Hamilton, 2020), propose that planning is construed as a self-regulatory strategy through which people put their intentions into practice. This volitional determinant can lead people to translate their risk perception into behaviors. Problem-focused coping includes actions, in which the main emphasis is placed on tasks or planning, and on attempts to solve problems (Mariani et al., 2020). Results such as those presented by Lin et al. (2020) show that the social cognition constructs with the largest effects on COVID-19 preventive behaviors were coping planning and action planning, both of which are considered instrumental coping strategies. Active and planning coping were associated with a better perceived general health and well-being (Chew et al., 2020), improved mental health (Jarego et al., 2021) higher global quality of life (Chwaszcz et al., 2021) and positive emotional state (Deepa and Manurali, 2021) during pandemic situations such as SARS and COVID-19. Furthermore, planning may also help people cope with lifestyle changes and facilitate compliance with health guidance (Sniehotta, 2009). A study on the role of coping strategies during a virus outbreak (the 2009 H1N1 flu pandemic) found that problem-focused coping was associated with a greater perceived risk of contagion and vaccination intentions among Canadian adults (Taha et al., 2013). Moreover, coping strategies have been found to be associated with self-efficacy (Flesia et al., 2020). Lowe et al. (2008) showed that people with high self-efficacy were more prone to use coping strategies to address specific problems. Nevertheless, the lockdown rules during the pandemic restricted people to their homes, a situation which may have threatened their sense of self-efficacy as their freedom to solve problems and create strategies was limited. Self-efficacy in an unpredictable and uncontrollable pandemic may play a significant role in determining the effect of instrumental coping on one's perceived ability to adhere to protection measures (Chong et al., 2020). However, the association between these variables has not been tested before.

An important factor that determines the use of protective behaviors is one's direct exposure to the event, or in this specific situation, having personally contracted COVID-19, or having a family member or close relation infected. According to Dryhurst et al. (2020), exposure to someone infected with the virus increased adherence to preventive behaviors against respiratory illnesses. These same authors concluded that people who had direct experience with COVID-19 (participants who reported they had tested positive for the virus, or suspected that they were infected) perceived more risk than those who did not have this experience. Most notably, in their study having personal and direct experience with COVID-19 was one of the most important predictors of engaging in protective measures. Galasso et al. (2020) also found that people with COVID-19 symptoms or who knew others with symptoms were more likely to comply with health measures than those who had no direct experience. However, Kim and Kim (2020) also concluded that knowing someone directly infected with COVID-19 did not predict action behaviors to prevent contagion.

Sex and age are important social determinants associated with health outcomes and practices. Galasso et al. (2020), with data from eight countries, show that when controlling for various sociodemographic variables and employment status, women were more likely than men to perceive the COVID-19 pandemic as very serious, be more supportive of restraining measures and adhere more to public health and social distancing measures. Niño et al.'s, results (2020) presented evidence to stress that males tended to be less fearful and perceived COVID-19 as less of a threat than females. Other studies have also found that males compared to females were more reluctant to adhere to protective measures to reduce their risk of contracting the virus (Coroiu et al., 2020; Shahnazi et al., 2020; Smith et al., 2020). These results concur with Bish and Michie's (2010) review on studies carried out on pandemics showing a consistent trend indicating that women were more likely to engage in protective behaviors, or Moran and Del Valle's (2016) meta-analysis reporting females as 50% more likely than males to get involved in health protective behaviors toward epidemic and pandemic respiratory infectious diseases.

Referring to age differences, Bish and Michie's (2010) review found that results were inconclusive, although mostly pointing toward an association between age and carrying out protective behaviors. Taylor (2019) stressed that young people are affected by an invulnerability bias that leads them to feel less at risk from suffering infectious diseases. This feeling of personal invulnerability intensifies risk-taking (Hill et al., 2012) and consequently inhibits engaging in protection measures. Niño et al. (2020) analyzing COVID-19 responses show that there was an age gradient in threat perceptions of coronavirus to personal health. Older aged participants perceived COVID-19 as a larger threat than younger aged participants did. Davies et al. (2020) also concluded that the older the respondents the greater the number of protective behaviors they adopted due to the existence of strong indications of age dependence in severity and mortality. A study conducted in 27 countries (Daoust, 2020) concluded that the 60+ age group is the most disciplined regarding all nine attitudes or measures of compliance with preventive rules and procedures toward COVID-19. This evidence suggests that variables such as gender or age may determine the adoption of self-protective measures.

### Research Aims

**Figure 1:**
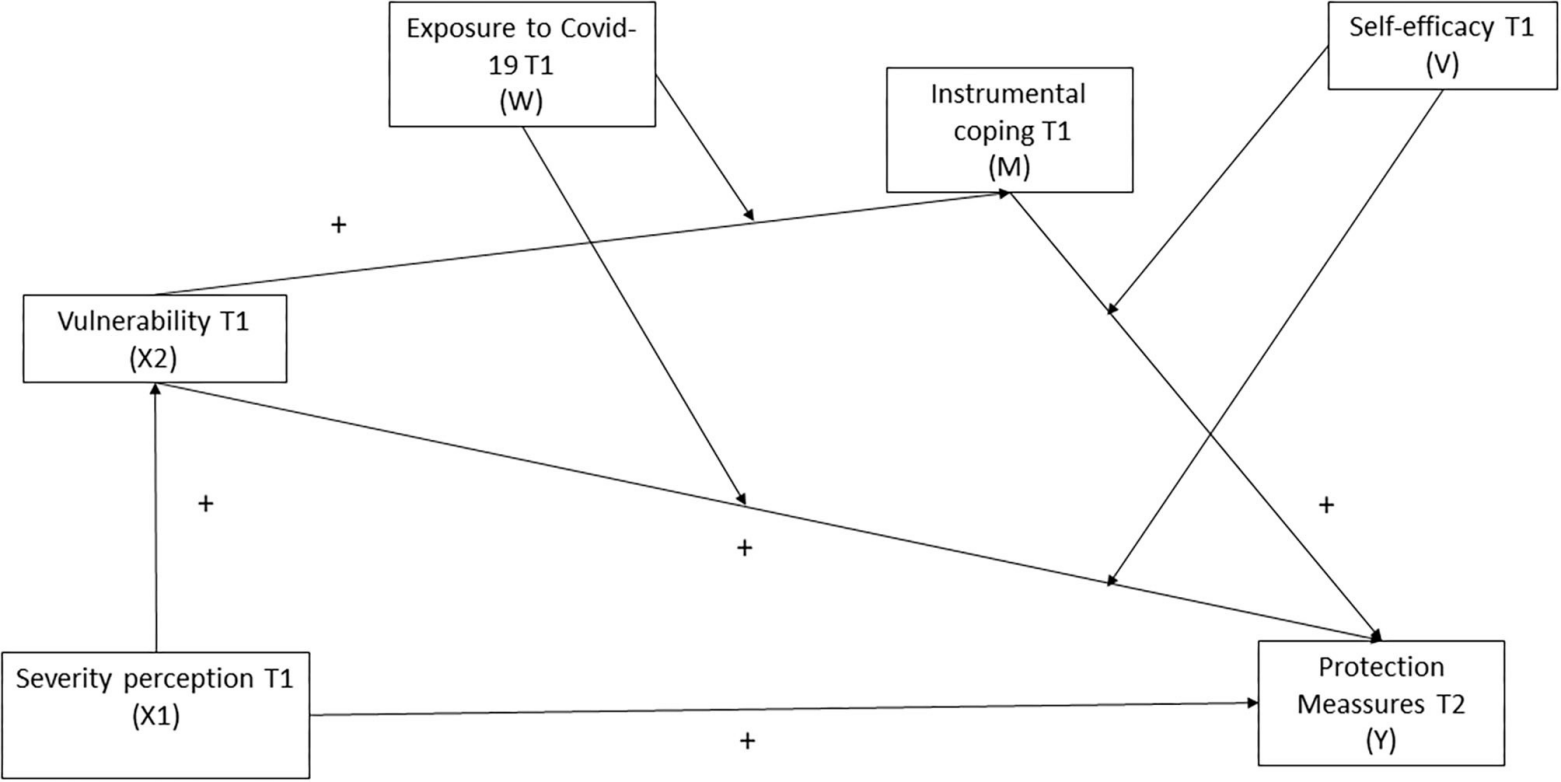
Moderated mediation theoretical integrated model depicting observed paths among study variables. Model equation defines one indirect effect(s) of X (perceived Risk, T1) on Y (Protection Measures T2), conditional on W (contact with Covid-19: no contact 0, contact 1) and V (Self efficacy: low self-efficacy 0 and high self-efficacy 1), and one direct effect of X2 on Y, conditional on W; and one direct effect of X1 on X2 and Y.

In the current study, we aimed to identify various factors that are most likely to influence adherence to the protective measures of COVID-19 outlined by the health authorities in Spain. We analyzed the predictive value of perceived severity and vulnerability of infection, self-efficacy and problem focused coping style for adhering to infection prevention behaviors during the first months of the COVID-19 pandemic. Specifically, this study aims:

- To test sex and age differences in perceived severity and vulnerability, self-efficacy, direct exposure to COVID-19, use of instrumental (planning and active) coping and adherence to behaviors to protect against contracting COVID-19 at T1 (March 2020) among a convenience sample of Spanish-speaking adults recruited during the first month of the lockdown imposed in Spain (March-April 2020).
- To examine the changes over the first month of the lockdown imposed in Spain (T1: March–T2: April 2020) in the proposed variables. Special attention will be awarded to the analysis of rates of adherence to specific protective measure recommendations toward COVID-19.
- To study the association between the proposed variables in T1 and engaging in protective measures in T2.
- To test a predictive integrated social cognition model analyzing how perceived disease severity, perceived personal vulnerability and self-efficacy (derived from Health Belief Model), and the use of instrumental coping strategies (derived from Coping Theory and the Health Action Process Approach) in a pandemic context (with direct exposure or experience to the disease) are related with future protection measures (see Figure 1). This model implies testing the following hypotheses:

Hypothesis 1: Perceived severity will be related to the individuals' vulnerability, this personal threat perception will be associated to instrumental coping and, at the same time, severity, vulnerability, and instrumental coping in T1 will be related to adherence to protection measures in T2.
Hypothesis 2: Instrumental coping in T1 will be a significant mediator between vulnerability in T1 and the adherence to preventive measures for COVID-19 in T2 (mediating effect).
Hypothesis 3: Self-efficacy and direct experience with COVID-19 (oneself, family or friends having being infected) in T1 will moderate the indirect effect (mediating effect) of instrumental coping between vulnerability in T1 and adherence to preventive measures for COVID-19 in T2. The combined moderating effects co-produced by direct experience and self-efficacy might indicate an interactive relationship or effect of these two psychological constructs affecting adherence to COVID-19 protective measures in T2.

## Materials and Methods

### Data Collection and Procedure

The longitudinal study was conducted from March 15 to 22 (first wave with 296 reported deaths) and April 15 to 25, 2020 (second wave: 21,717 reported deaths) (Spanish Ministry of Health, 2021). Data was collected during the lockdown enforced in Spain, as during that time-period (March-April), restrictions on daily life were applied to all citizens (Boletín Oficial del Estado, 2020). These two periods not only reflect the increasing number of deaths and infections, but also an evolution from one of the strictest lockdowns in Europe to the gradual relaxation of some of the toughest measures (e.g., as from mid-april people were allowed to leave home in more circumstances). Participants were asked to complete a series of online questionnaires measuring COVID-19 severity and vulnerability perceptions, direct exposure to COVID-19, perceived self-efficacy, instrumental coping, and use of protection measures.

For data collection, and due to the impossibility of physical contact, the survey was hosted on the Qualtrics platform and distributed via snowball convenience sampling through university press releases, the co-author's professional and personal networks (e-mail lists) and various social media accounts (e.g., Twitter, Facebook). Eligibility criteria were having sufficient Spanish-language skills and being 18 years of age or older. Each person was assigned a unique identifier when he/she completed the first wave of the survey. Participants who had granted permission were contacted in subsequent waves of the survey using this unique identifier to pair the responses of the two waves. Participants took an average of 40 min to respond the questionnaire.

Participation in the study was voluntary and individuals provided online informed consent by using a tick box on the survey and acknowledging that they had read and understood the conditions of their participation in the survey. The Bioethics Committee of the University of Burgos approved the research and its implementation (IR10/2020) following the recommendations of the Declaration of Helsinki concerning research carried out with human participants.

### Measures

#### Demographic Questionnaire

The Demographic Questionnaire was developed by the researchers to gather information regarding a series of participants' sociodemographic characteristics such as sex, age, educational level, and relationship status.

#### Perceived Disease Severity

An *ad hoc* measure consisting of one item was created. Participants were asked “To what degree do you think coronavirus is a major or serious disease?” rated on a 7-point Likert scale (1 = not at all serious, to 7 = very serious).

#### COVID-19 Perception of Vulnerability

This consisted of an *ad hoc* measure composed by an item regarding perception of vulnerability based on Protection Motivation Theory (Rogers, 1975, 1983) (“Coronavirus is a real threat to you”) rated on a 7-point Likert scale (1 = no threat at all, to 7 = very high threat).

#### Instrumental Coping

An adapted version of the Emotional Regulation Scale (MARS) (Larsen and Prizmic, 2004; Puente-Martínez et al., 2018) was used to measure the frequency of use of the instrumental strategy to cope with COVID-19. This specific strategy was measured by two-items (“Making a plan to deal with what happened and be able to do something to change the situation” and “Acting or doing something to improve or solve the problem or situation that caused my mood”) rated on a Likert scale from 0 (never) to 6 (always). Higher scores indicate a greater use of this way of confronting COVID-19. Internal consistency was α = 0.76 in T1 and T2.

#### Self-Efficacy

Following recommendations by Bandura (1997), a self-efficacy scale was created so that items would coincide with the specific nature of the problem and situation. It consists of three items assessing the ability to comply with the protection measures against COVID-19 put forward by the authorities (i.e., “Are you able to comply successfully with all the protective measures indicated by the authorities even though it may affect your everyday activities or be troublesome”). The respondents were asked to indicate on a 7-point Likert-type scale their level of agreement or disagreement with each statement (“1 = Totally disagree” to “7 = very strongly agree”). Internal consistency was α = 0.82 in T1 and α = 0.87 in T2.

#### Direct Exposure to COVID-19

An *ad hoc* scale was created to attest direct contact or exposure to COVID-19. It consists of three items measuring if oneself, close relatives (partner, father, mother, brother, son, daughter, grandparents, etc.) or friends have contracted COVID-19. A dichotomous variable was created (0 = No exposure, 1 = Yes exposure). Participants who indicated at least one “yes” were considered to have had a direct experience with COVID-19.

#### Use of Protection Measures

This was an *ad hoc* scale of protection measures based on the recommendations given during the first weeks of March 2020 by the Spanish Ministry of Health (http://www.mscbs.gob.es). The measure included 7 items in the first wave (T1) and nine items in the second wave (T2). This increase was due to the inclusion of new recommendations given by the Ministry of Health in April. For the comparison between both waves only the first seven items (i.e., “Wash hands frequently with soap and water,” “Keep more than 1-m distance with other people,” “Cover nose and mouth with a handkerchief when coughing”) were considered. For analyses conducted in T2 the full nine items were included (including the two new measures “Use sanitary gloves when leaving home,” “Use facemask when going outdoors”). Respondents were asked if they had adopted each of the protective behaviors (0 = no, 1 = yes). The sum frequency of use was calculated, where higher scores indicate a greater use of protection measures. Reliability analysis showed a Cronbach reliability in T1 α = 0.53 and in T2 α = 0.56.

#### Data Analysis

Demographic data and test scores of participants were summarized by descriptive and frequency statistics (means, standard deviations, frequencies, and percentages). Student's *t*-test for independent samples, chi square test and correlation analyses were conducted on the scores in T1 to determine attrition, sex and age differences (Objective 1). A series of General Lineal Models were performed to test differences in T1 and T2 in the variables under study controlling for sex and age (Objective 2). Effect sizes of the mean differences were estimated using Cohen's d (Cohen, 1988) criteria. A small effect was conceptualized as *d* = 0.20, medium *d* = 0.50, and large *d* = 0.80. To analyze the relationship between the study's variables, partial correlations (*r*p) were conducted including age, sex and protection measures in T1 as control variables (Objective 3).

Finally, we analyzed the performance model in two steps using Mplus statistical software (Version 8.5, Muthén and Muthén, 2017). First, to examine associations between the variables of interest (Hypothesis 1) and evaluate the mediating role of instrumental coping (Hypothesis 2), path analyses were conducted at baseline (T1) and a month later (T2) controlling for sex, age, and adherence to COVID-19 protection measures in T1. A variety of global fit indices were used to determine whether the data fitted the proposed path model, including a chi-square test of model fit (χ^2^), the root mean square error of approximation (RMSEA; value should be <0.08 to declare satisfactory fit), the comparative fit index (CFI; value should be >0.90), the TuckerLewis index (TLI; value should be >0.90), and the standardized root mean square residual (SRMR; should be <0.05) (Kline, 2010). Indirect effects were calculated using 10,000 bootstrapping samples, generating confidence intervals of the bias-corrected bootstrap type (BCBootstrap). A conditional indirect effect is considered statistically significant if the confidence interval (CI at 95%) does not include the value 0. All scores were standardized previous to performing the analyses.

Second, we integrated the proposed moderator variables (self-efficacy and exposure to COVID-19 in T1) into the model and empirically tested the overall moderated mediation hypothesis (Hypothesis 3). Self-efficacy was construed as a dummy variable (0 = low self-efficacy; 1 = high self-efficacy) based on the mean scores. We tested the indirect effects including each moderator separately. Then, a pair of two-way interactions were used to test moderation in the path model along with the main effects: X2^*^W: Perceived vulnerability ^*^ contracting COVID-19 (W1 = 0: no direct exposure COVID-19 vs. W2 = 1: direct exposure COVID-19) and M^*^V: instrumental coping ^*^ self-efficacy (V1 = 0: low self-efficacy and V2 = 1: high self-efficacy). Hence, assuming this moderation hypothesis receives empirical support, it is plausible to assume that the strength of the hypothesized indirect effect (mediation) is conditional on the value of the moderators (exposure, or not, to COVID-19 and low/high perceived self-efficacy) when controlling for sex, age, and protection measures in T1.

#### Participants

A total of 1220 participants completed the questionnaire during the first wave (T1), of which *N* = 757 also completed the second wave (T2). The sample is non-representative of the general Spanish population because there is a larger proportion of females and tertiary educated participants in the study than the national average. Also due to the imposed lockdown only respondents with internet connection could answer the survey. Moreover, although participants lived in all 17 autonomous communities and in one of the two autonomous cities in which the country is administratively organized they were not a stratified representative sample of each of these communities.

## Results

### Descriptive Statistics

Attrition analyses were performed to determine whether participants included in this study (those who participated in both waves) differed from the dropouts (*n* = 463) with respect to their baseline levels on the study's variables. *T*-test results show that there were only differences in the perceived self-efficacy measure between participants and dropouts although the effect size is small (see Table 1). Cross tabulation results showed that the two samples did not differ regarding sex [χ${}_{\left(1220,\;1\right)}^{2}$ = 0.525, *p* = 0.469] or contracting COVID-19 (oneself, family or friends) [χ${}_{\left(1220,\;1\right)}^{2}$ = 2.522, *p* = 0.112].

**Table 1:** Attrition descriptive results.

	**Participants** ***n*** **=** **757**		**Dropouts** ***n*** **=** **463**				
**Variables in T1**	***M***	***SD***	***M***	***SD***	***t***	***p***	***d***
Age	38.69	12.98	37.35	13.5	−1.72	0.086	0.10
Severity	5.20	1.42	5.29	1.40	1.10	0.270	0.06
Vulnerability	4.23	1.75	4.26	1.83	0.25	0.800	0.02
Instrumental coping	3.07	1.50	3.20	1.51	1.51	0.131	0.09
Self-efficacy	17.39	3.53	16.63	4.07	−3.33	0.001	0.20
Protection measures	5.44	1.36	5.47	1.39	0.45	0.651	0.02

The sample consists mainly of women, highly educated participants, and who either have a partner or are single (see Table 2). The mean age was 38.69 (*sd* = 12.98, range 18–77 years old).

**Table 2:** Participant demographics characteristics.

	***N* = 757**	***%***
**Sex**
Male	195	25.8
Female	562	74.2
**Civil status**
Married	274	36.2
Civil Partnership/Cohabiting	221	29.2
Single	223	29.5
Divorced/Separated	29	3.8
Widowed	8	1.1
Other	2	0.3
**Education**
Primary Education	22	2.9
Secondary Education	180	23.8
Higher or Tertiary Education	345	45.5
Post Tertiary Education (Master/Ph.D)	210	27.8

### Differences in Variables According to Sex and Age

T student contrasts showed that women use the instrumental coping strategy more frequently, perceive themselves as having more self-efficacy and comply more with the protective measures (in T1 and T2) than men (see Table 3). Effect sizes were small. There are no significant sex differences in perceived severity or vulnerability, or in having contracted the virus themselves, their close relatives or friends [χ${}_{\left(757,\;1\right)}^{2}$ = 0.286, *p* = 0.593].

**Table 3:** Differences according to sex.

	**Men** ***n*** **=** **195**		**Women** ***n*** **=** **562**		
	***M***	***SD***	***M***	***SD***	***t***	***p***	***d***
Severity T1	5.04	1.50	5.25	1.39	−1.81	0.071	0.14
Vulnerability T1	4.37	1.70	4.19	1.77	1.26	0.207	0.10
Instrumental coping T1	2.76	1.55	3.17	1.47	−3.34	0.001	0.27
Self-efficacy T1	16.93	3.78	17.55	3.43	−2.14	0.033	0.17
Protection measures T1^a^	5.10	1.42	5.56	1.32	−4.11	0.0001	0.34
Protection measures T2^b^	6.25	1.89	6.90	1.63	−4.29	0.0001	0.37

a *Protection measures at T1 range from 0 to 7;*

b *ranged from 0 to 9*.

Age is positively and significantly associated with severity (*r* = 0.21, *p* = 0.0001) and vulnerability in T1 (*r* = 0.31, *p* = 0.0001) and use of protection measures in T1 (*r* = 0.18, *p* = 0.0001) and T2 (*r* = 0.20, *p* = 0.0001). It was not associated with use of instrumental coping (*r* = −0.07, *p* = 0.073), self-efficacy (*r* = 0.03, *p* = 0.484) or having contracted the virus themselves, their close relatives or their friends [*t*_(755)_ = 1.33, *p* = 0.183].

### Differences in Variables Between Time 1 and Time 2

General Lineal Models controlling for sex and age revealed that participants reported higher perceived severity and greater use of protective measures at T2 than at T1. Effect sizes are small (Table 4). Moreover, McNemar chi-square results showed that participants in T1 had less direct contact with COVID-19 (oneself, family or friends) than in T2 (χ^2^ = 200.29, *p* = 0.0001). The percentage of people who had direct experience rose from 11.76% (*n* = 89) in T1 to 41.74% (*n* = 316) in T2.

**Table 4:** Differences from Time 1 and Time 2 for variables under study.

	**Time 1**		**Time 2**		
	***M***	***SD***	***M***	***SD***	***F***	***p***	***d***
Severity	5.20	1.42	5.53	1.32	58.78	0.0001	0.24
Vulnerability	4.23	1.75	4.27	1.71	0.39	0.531	0.02
Instrumental coping	3.07	1.50	3.01	1.48	1.19	0.276	0.04
Self-efficacy	17.39	3.53	17.33	4.00	0.16	0.690	0.02
Protection measures^a^	5.44	1.36	5.74	1.29	56.68	0.0001	0.23

a *Range from 0 to 7. Covariates: sex and age*.

The percentage of use of the different protection measures vary between almost 99% (Avoid close contact with people infected with coronavirus) and 41% (Wearing facemasks when leaving home). There is a significant increase over time in three of the protection measures, while none suffer a decrease in their use. The most important increase occurs in washing and disinfecting objects and surfaces that are frequently touched or manipulated (Table 5).

**Table 5:** Differences from Time 1 and Time 2 in protection measures.

**Variables**	**T1**	**T2**	**χ^**2**^*McNemar***
	**Yes (%)**	**Yes (%)**	
Avoid close contact with people infected with coronavirus	98.9	98.2	0.842, *p* = 0.359
Avoid touching one's eyes, nose or mouth without washing one's hands	81.2	85.5	7.87, *p* = 0.005
Frequently wash one's hands with soap and water for at least 20 s	92.1	92.5	0.068, *p* = 0.795
Use hand sanitizer containing at least 60% alcohol if there is no soap and water	63.4	67.2	4.53, *p* = 0.033
Cover one's nose and mouth with a handkerchief when coughing or sneezing and later throw it in a dustbin	69	72.5	3.29, *p* = 0.069
Wash and disinfect objects and surface that are frequently touched or manipulated	47.6	64.6	73.47, *p* = 0.0001
Keep a distance of at least 1 m when interacting or talking to other people	91.7	93.8	3.516, *p* = 0.060
Use sanitary gloves when leaving home		57.9	
Wearing face masks when leaving home		41.2	

### Relationship Between Variables

All partial correlations when controlling for sex, age, and protection measures in T1 are presented in Table 6. Vulnerability correlated with instrumental coping in T1. In addition, use of protection measures in T2 correlated significantly with more severity, vulnerability, more use of instrumental coping and more self-efficacy. Moreover, there were no differences between having contracted the virus themselves, their close relatives or their friends or not, and engaging in protection measures [*t*_(755)_ = −0.368, *p* = 0.713).

**Table 6:** Relationship between variables.

	**1**	**2**	**3**	**4**	**5**
1. Severity T1	-				
2. Vulnerability T1	0.58^***^	-			
3. Instrumental coping T1	0.06	0.08^*^	-		
4. Self-efficacy T1	−0.01	−0.028	0.05	-	
5. Protection measures T2	0.14^***^	0.14^***^	0.11^**^	0.07^*^	-

*** *p ≤ 0.001*,

** *>p ≤ 0.010*,

* *p ≤ 0.050*.

### Path Model

**Figure 2:**
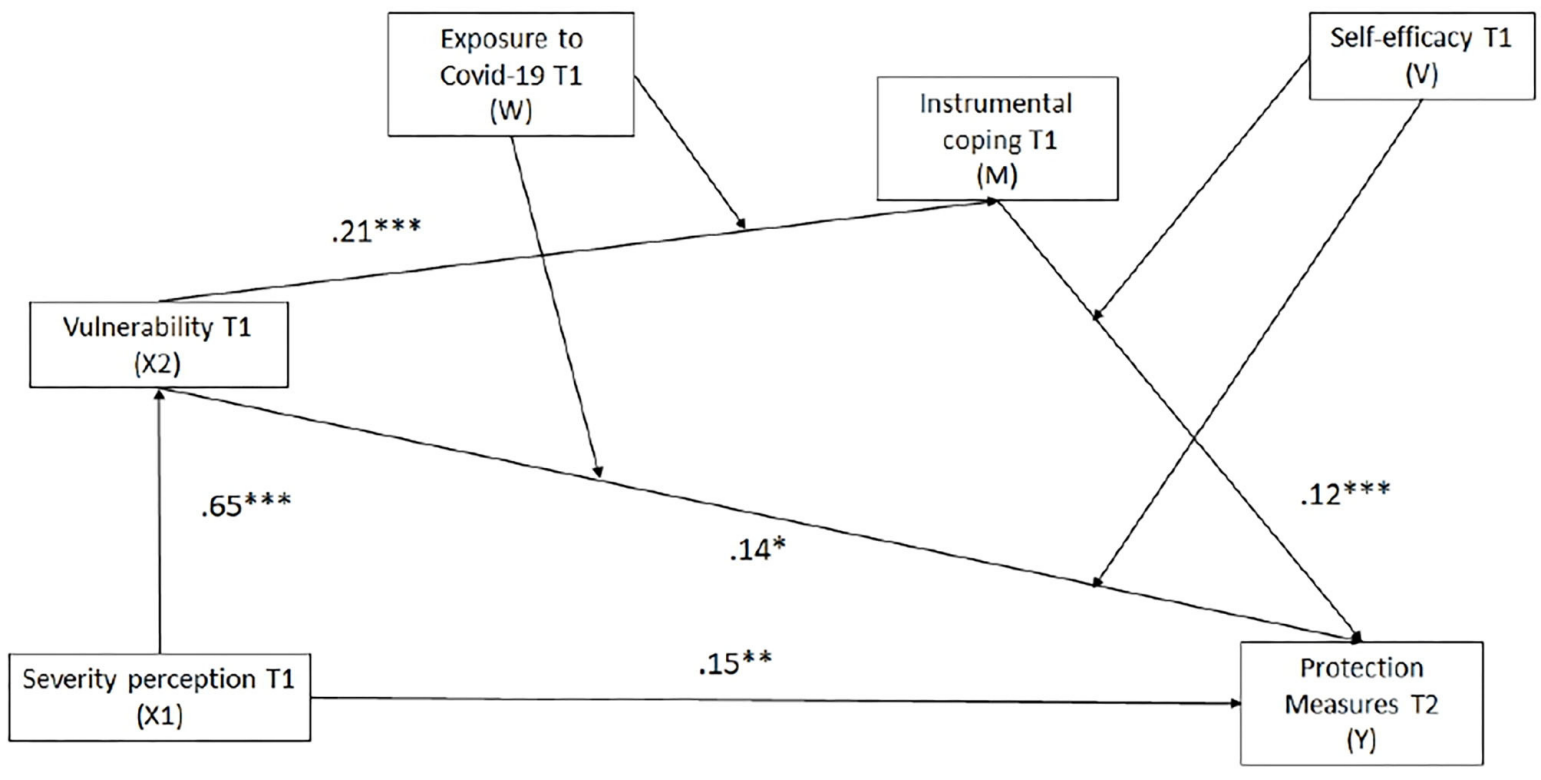
Estimated standardized path coefficients for proposed model. *0.05, **0.01, ***0.001.

A path analysis was used to test the theoretical model outlined in Figure 1. The hypothetical model provided a good fit to the data (χ^2^ = 6.59, df = 4, *p* = 0.159; RMSEA = 0.03, 95% CI = 0.00, 0.07; SRMR = 0.02; CFI = 0.99, TLI = 0.99) suggesting that the observed data matched well with the proposed path model. The hypothetical model accounted for significant variance in the use of protection measures at T2 or *R*^2^ = 0.35 (see Figure 2). Therefore, statistical significance of direct and indirect effects of the model were examined to analyse the results of hypothesis testing.

Results showed a direct and significant relationship between perceived severity and vulnerability to contract COVID-19 at baseline (X1 → X2). Severity is associated with an increase in the use of protection measures at T2 (X1 → Y). Moreover, vulnerability of contracting COVID-19 increased the use of instrumental coping strategies (X2 → M) and was associated with a greater use of protection measures in T2 (X2 → Y). Instrumental actions were also related to a higher adherence to COVID-19 protection measures at T2 (M → Y).

In addition to direct effects, indirect effects indicated that the relationship between severity (X1 → X2 → M → Y) (Indirect effect: *b* = 0.01, Se = 0.01, *p* = 0.043) and vulnerability of contracting COVID-19 (X2 → M → Y) (Indirect effect: *b* = 0.012, Se = 0.01, *p* = 0.042) at T1 and the use of protection measures at T2 was mediated by instrumental coping. Thus, individuals who reported to perceive higher vulnerability tended to use more instrumental coping to deal with the situation, which, in turn, was associated with more use of protective measures at T2.

Additionally, a moderated mediation examined whether the indirect effect of vulnerability on the use of preventive measures for COVID-19 through instrumental coping would be moderated by direct exposure to COVID-19 and perceived self-efficacy (see Table 7). Hence, we examined four conditions to establish whether the strength of the mediation via instrumental coping differs across various levels of exposure to COVID-19 (W1 = 0: no direct exposure to COVID-19; W2 = 1: direct exposure to COVID-19) and perceived self-efficacy (V1 = 0: low self-efficacy, V2 = 1: high self-efficacy).

**Table 7:** Results of the moderated mediation analysis.

**Predictors**				**95% CI**	
	***B***	***SE***	***p***	***LL***	***UL***
	**Independent variable: Vulnerability (X2)**					
Sex	−0.18	0.07	0.009	−0.348	−0.001
Age	0.14	0.03	0.0001	0.062	0.211
Protection measures-T1	0.09	0.03	0.002	0.014	0.163
Severity (X1 → X2)	0.65	0.03	0.0001	0.578	0.716
	**Mediator: Instrumental coping (M)**					
Sex	0.30	0.13	0.023	0.034	0.650
Age	−0.16	0.06	0.009	−0.313	−0.005
Protection measures-T1	0.17	0.06	0.003	0.024	0.318
Vulnerability (X2 → M)	0.21	0.05	0.0001	0.067	0.343
COVID-19 (W)	0.09	0.05	0.064	−0.035	0.222
Vulnerability × COVID-19 (X2*W → M)	−0.06	0.06	0.320	−0.198	0.094
	**Dependent variable: Protection Measures (Y)**				
Sex	0.20	0.05	0.0001	0.067	0.343
Age	0.19	0.06	0.001	0.038	0.324
Protection measures T1	0.65	0.03	0.0001	0.578	0.716
Severity-T1 (X1 → Y)	0.15	0.06	0.009	0.015	0.319
Instrumental coping-T1 (M → Y)	0.12	0.03	0.0001	0.034	0.207
Self-efficacy-T1 (V → Y)	0.17	0.12	0.150	−0.132	0.470
Instrumental coping × self-efficacy (M*V → Y)	−0.04	0.03	0.961	−0.086	0.084
Vulnerability T1 (X2 → Y)	0.14	0.07	0.031	0.024	0.312
COVID-19	0.01	0.05	0.768	−0.100	0.126
Vulnerability × COVID-19 (X2*W → Y)	0.15	0.05	0.001	0.032	0.274
Vulnerability T1 × self-efficacy (X2*V → Y)	−0.04	0.05	0.426	−0.166	0.086
**Conditional indirect effects for each value of the moderator**					
Vulnerability × no exposure COVID-19 (X2 → W1 → Y)	0.02	0.01	0.009	0.006	0.054
Vulnerability × exposure COVID-19 (X2 → W2 → Y)	0.02	0.01	0.095	−0.004	0.054
Instrumental coping × low self-efficacy (M → V1 → Y)	0.03	0.01	0.009	0.006	0.054
Instrumental coping × high self-efficacy (M → V2 → Y)	0.06	0.03	0.046	0.001	0.154
**Conditional total effects for each value of the moderator**					
X2 → W1 → Y	0.16	0.07	0.012	0.002	0.337
X2 → W2 → Y	0.16	0.07	0.016	0.009	0.328
M → V1 → Y	0.16	0.07	0.012	0.002	0.337
M → V2 → Y	0.20	0.07	0.006	0.017	0.393
**Conditional direct effects for each combination of moderator values**					
Vulnerability x no exposure COVID-19 × low self-efficacy (X2*W1*V1)	0.14	0.07	0.031	0.024	0.312
Vulnerability x exposure COVID-19 × low self-efficacy (X2*W2*V1)	0.29	0.08	0.0001	0.093	0.496
Vulnerability x no exposure COVID-19 × high-self efficacy (X2*W1*V2)	0.10	0.08	0.214	−0.104	0.315
Vulnerability x exposure COVID-19 × high self-efficacy (X2*W2*V2)	0.25	0.10	0.009	0.001	0.496
**Combined conditional Indirect effects of Vulnerability on Protection Measures**					
X2 → W1 × V1 → Y	0.03	0.01	0.009	0.006	0.054
X2 → W2 × V1 → Y	0.02	0.01	0.095	−0.004	0.054
X2 → W1 × V2 → Y	0.02	0.01	0.037	0.001	0.064
X2 → W2 × V2 → Y	0.02	0.01	0.139	−0.003	0.062
**Combines conditional total effects for each combination of moderator values**					
X2 → W1 × V1 → Y	0.17	0.07	0.012	0.002	0.337
X2 → W2 × V1 → Y	0.31	0.08	0.0001	0.114	0.507
X2 → W1 × V2 → Y	0.12	0.08	0.125	−0.008	0.335
X2 → W2 × V2 → Y	0.27	0.10	0.005	0.016	0.511

*CI, confidence interval; LL, lower limit; UL, Upper limit; Control variables: sex, age, and protection measures in T10*.

First, we analyzed the moderation effects of having direct exposure to COVID-19 and self-efficacy. Results showed non-significant direct interactions between perceived vulnerability and exposure to COVID-19 on instrumental coping (X2^*^W → M) and also between instrumental coping and self-efficacy on the adherence to protection measures at T2 (M^*^V → Y). However, the interaction between vulnerability and COVID-19 direct exposure on protection measures is significant (X2^*^W → Y) and non-significant for self-efficacy (X2^*^V → Y).

Then, we tested whether the conditional indirect effect of perceived vulnerability on protection measures via instrumental coping was different for people who had direct experience or not with COVID-19 and low or high self-efficacy. For the COVID-19 exposure moderator, the conditional indirect effect of perceived vulnerability of contagion was only significant in the non-exposure condition (X2 → W1 → Y). In other words, participants who did not have a direct experience with COVID-19 increased the effect of vulnerability on protection measures via the use of instrumental coping strategies. Regarding the self-efficacy moderator, the indirect conditional effect was statistically significant for the low and high self-perceived self-efficacy conditions (M → V1 → Y and M → V2 → Y). These results suggest that the effect of vulnerability on the use of protection measures in T2 through instrumental coping was strengthened in both self-efficacy conditions.

Combined conditional indirect effects of vulnerability on protection measures (X2 → WV → Y) showed significant indirect effects only in the conditions of not having direct experience with COVID-19 x low self-efficacy (X2 → W1^*^V1 → Y) and not having direct experience with COVID-19 x high self-efficacy (X2 → W1^*^V2 → Y).

## Discussion

In a pandemic, individual decisions that affect both oneself and the community as a whole are as important as the decisions a government may try to implement. This study analyzes the influence of socio-cognitive factors such as perceived severity and vulnerability, self-efficacy, coping strategies and direct exposure to COVID-19 measured at the beginning of a lockdown (baseline scores) on adherence to protection measures for COVID-19 a month later while taking into account participant's sex, age and the previous use of protection measures.

As regards sociodemographic variables, results confirm that females perceived higher levels of self-efficacy, used more instrumental coping and more protection measures than males. This is consistent with the literature indicating sex differences in responses to COVID-19, especially the adoption of precautionary measures (Bish and Michie, 2010; Coroiu et al., 2020; Galasso et al., 2020; Niño et al., 2020; Shahnazi et al., 2020; Smith et al., 2020). There were no differences in perceived severity and vulnerability between sexes. Results from a meta-analytic review show that perceived severity of a disease may depend on other non-personal factors such as the proximity of the study population, high risk areas, information, or even the phase of the pandemic in which surveys were administered (Moran and Del Valle, 2016). Results confirmed that age was positively associated with higher perceptions of perceived disease severity, personal vulnerability, and the use of protection measures. Various studies have found that older age is related with a higher perception of severity and mortality (Davies et al., 2020) and with more use of preventive measures (Storopoli et al., 2020). Congruently, a study concluded that a sense of invulnerability is more common among young people since older adults tend to perceive the virus as more threatening (Taha et al., 2013). Therefore, the results of this study are consistent with the widespread idea that adolescents and emerging adults may engage in risky behavior, or at least in less protective measures, in part because of their sense of invulnerability to injury, harm, and danger (Lapsley and Hill, 2010) (Objective 1).

Findings reveal a general increase in the use of protective measures over time, although the effect size is small (objective 2). In this sample, social distancing (e.g., keep a distance of at least 1 m and avoid contact with people infected) and washing hands were the most frequent preventive behaviors (>90% in T1 and T2), while the two measures included in the Government's recommendations in T2 after a month in lockdown were less used (e.g., use sanitary gloves: 58%, and wearing face masks in public: 41%). These results may suggest that despite measures taken to inform the public of the need to engage in protective measures, some of these, that may be perceived as strongly interfering with everyday interactions, elicit a stronger backlash questioning their efficacy. Nevertheless, in general participants complied with many of the protection measures suggested by health officials.

In addition, results revealed that participants increase their perception of severity and the use of protective measures over time. A possible explanation is that in addition to a greater perceived severity during the first wave of the pandemic (the number of people dying during this month increased dramatically) the knowledge about the virus was at first limited and the use of some protective measures controversial because there were doubts on their efficacy to reduce the infection. For instance, the use of facemasks in public settings was not supported by government officials until after more than a month of the start of the pandemic in Spain. The crescent scientific evidence supporting the effectiveness of different measures to avoid contagion may have increased the use of more protective measures. Regarding personal vulnerability there may be various reasons that could explain why there were no significant changes over time. First, the fact that the population was confined in strict lockdown and all but essential outgoings were prohibited coupled with the adoption of protective measures may have increased the sense of control. Second, recent experiences with other types of pandemics may have had an impact on people's beliefs about the threat of SARS-CoV-2. For example, the SARS outbreak of 2003 was overcome with relative ease. The virus spread rapidly in 30 countries but was contained in ~6 months. This experience could have led to an underestimation of the dangers of the new SARS-CoV-2 virus despite official warnings (Bottemanne et al., 2020). Third, this result could also be related to the cognitive bias of optimism, that is, the underestimation of the possibilities of experiencing negative health events compared to others (Weinstein, 1980). In this study, although both severity and perceived vulnerability are high, people rate their personal vulnerability to contracting COVID-19 as lower compared to the overall threat it poses. Previous studies have confirmed in different countries (Italy and Romania: Druicǎ et al., 2020) (Germany, UK and the USA: Kuper-Smith et al., 2020) (France, Italy, Switzerland and United Kingdom: Raude et al., 2020a) the existence of an optimism bias in the context of COVID-19. Sharot (2011) suggests that optimism, provided it is not excessive, is vital for physical and mental health, and thus this misbelief would have an adaptive function. In addition, this bias increased over time, probably because the initial confusion gave way to a situation of uncertainty affecting the subjective beliefs of rational people about their possibility of contagion (Stout, 2012) (Objective 2).

In the correlation analyses, self-efficacy is not associated with the perception of severity and vulnerability and is the variable most weakly associated with the adoption of protection measures. Social cognitive theory subscribes that human functioning is a product of the interplay of intrapersonal influences (self-efficacy), the behavior individuals engage in, and the environmental forces that impinge upon them (Bandura, 2012). Under imposed social and physical constraints, individuals are disinclined to act on their self-efficacy beliefs. Individual self-efficacy mainly influences what people can directly control. However, in a pandemic situation, the success of individual actions does not depend only on the belief in one's own capabilities but also on collective efficacy (Stajkovic et al., 2009) (Objective 3).

Our path model provides useful information about the psychological pathways of behaviors in controlling or preventing the spread of the COVID-19 infection and in complying with the recommendations dictated by authorities. We found that COVID-19 symptom severity increased awareness of the hazards and personal risks of harm derived from COVID-19. Perceived severity and vulnerability significantly predicted adherence behaviors to protection measures. Previous studies also mention that perceived vulnerability is an important determinant of the people's willingness to cooperate and adopt health-protective behaviors during COVID-19 (Chong et al., 2020). Supporting this result, various studies found that perceived vulnerability or personal understanding of the disease and its consequences may influence psychological and behavioral responses (Sawyer et al., 2019; Malecki et al., 2021). This phenomenon is also confirmed in our results suggesting that instrumental coping is positively associated with adherence to protection measures (Hypothesis 1).

It is interesting to note that planning and direct problem-solving coping have shown to play a mediating role on vulnerability and protection adherence behaviors. This indicates that at the beginning of the COVID-19 pandemic, people's abilities to formulate or engage in instrumental coping strategies increased the effect of perceived vulnerability on engaging in protective measures. These findings are consistent with both Lin et al.'s (2020) results and Chong et al. (2020) who report that people might choose to adopt problem-focused coping to manage the vulnerability concerning the infection risk and impact of the COVID-19 outbreak, thus positively having an impact on their adherence behaviors (Hypothesis 2).

Consistent with hypothesis 3, the conditional indirect effects showed that the effect of vulnerability on the use of protection measures at T2 through the use of the instrumental coping strategy was strengthened in participants with both perceived high and low self-efficacy who had not been exposed to COVID-19. Instrumental coping or planning is a strategy that facilitates the task and is related to how individuals prepare to perform a behavior. These plans could help anticipate certain obstacles, increasing the effect of vulnerability on the adoption of preventive or protective measures (Lin et al., 2020). Based on these results, the strategy of active instrumental self-regulation or planning in the volitional phase that determines the subsequent enactment of the target behavior seems to be necessary only in the case of those who have not been directly exposed to the virus regardless of their level of self-efficacy. The scale of the COVID-19 pandemic is unprecedented in modern times and there remain doubts over the efficacy of protective behaviors. In fact, even though people may feel confident in their own ability to engage in protective behaviors, they do not necessarily think that their response is efficient in reducing the threat (Tang et al., 2020). Moreover, this result may be explained since self-protective measures (e.g., hand-washing, avoiding public places, wearing face-masks, social distancing) have been imposed by governmental policies, and self-efficacy is actually only monitoring compliance with these norms.

Regarding direct exposure, several reasons could justify why the effect of the active instrumental strategy is not an effective mediator in the case of people who have had direct experience with COVID-19. First, direct experience may provide information about the disease and the actual effectiveness of the adopted preventive measures (Weinstein, 1989). Second, personal experiences may be easier to remember and more likely to be recalled at appropriate times to stimulate action (Fazio et al., 1978). Third, information elicited from personal experience generates less uncertainty than when it is evoked in other ways, so such information may be more compelling and produce more stable cognitions (Doll and Ajzen, 1992). Fourth, personal experience with this negative event can lead to fear of recurrence and people may act to reduce unpleasant feelings of fear (Leventhal et al., 1983). In Harper et al.'s (2020) study, the only predictor of positive behavioral change (e.g., social distancing, improved hand hygiene) was fear of COVID-19. Therefore, people who have had direct experience with COVID-19 may not need to resort to prior preparation or planning to reinforce the adoption of preventive behaviors. Perceived vulnerability may translate directly into greater adoption of prevention measures with no necessary intermediate variable.

A series of limitations of the current study must be acknowledged. First, the data was collected from the digital space due to the conditions derived from the total lockdown caused by the disease; hence, it did not allow for random sampling to select individuals, nor are they representative of the general Spanish population although there are representatives from each autonomous region in the country. Nevertheless, as Balanzá-Martínez et al. (2020) mention, in this pandemic behavioral medicine may benefit from surveys carried out remotely to reach a larger number of individuals in need and generate quick and effective data to inform policymakers. Second, although data analysis showed only slight differences in sociodemographic characteristics, there is an important homogeneity of sample features (i.e., 74% female, >70% completed secondary education), which might affect the generalizability of our findings to predominantly male and more diverse samples, or individuals without easy access to the internet and social media platforms (Facebook, Twitter). Third, results are based solely on self-report with the problem of susceptibility to social desirability bias. Future studies could benefit from, for example, using a diary-based design to measure changes across time. Fourth, due to the period in which the study was conducted, and the spread of the virus, the number of possible participants directly affected by COVID-19 was low (*n* = 6). Due to this, the direct exposure measure was created by including if oneself, a family member, or friend (not acquaintance) was suffering the disease. However, there is a large imbalance in the number of people who had direct contact with the virus or not (~12–88% in T1) that lead to being cautious with the results. Nevertheless, studies such as Guo et al. (2020) have used the same analytical strategy. In this study, we did not analyze other variables that could influence the use of instrumental coping or adherence to protection measures such as work status or previous illness (Albert and Duffy, 2012). For instance, a study found that health workers were significantly less risk-averse compared to non-healthcare workers (Galandra et al., 2020). Moreover, personality traits such as sensation seeking, impulsivity, anxiety sensitivity (DeGrace et al., 2021) or dark triad traits could lead to less compliance with pandemic restrictions or exhibit less prevention (Nowak et al., 2020; Zajenkowski et al., 2020). Finally, certain medical conditions or chronic illness, and higher risk of contracting severe COVID-19 may also associate with a greater adherence to protective measures (Meier et al., 2020) and in consequence may affect our results.

Despite these limitations, our study makes an important contribution to the understanding of the factors associated with the adherence to protective behaviors during the pandemic. Moreover, this study captures the changes in participants' perceptions of an unprecedented event such as a global pandemic and total lockdown by measuring shorter timeframes that may be more temporally precise with respect to disruptions caused by the pandemic and the important social and legal changes that took place in such a short period. These results are not only theoretically sound, but also have practical implications. Based on evidence extracted from this study, health interventions should consider strategies that target change in perceived severity and vulnerability and enhance instrumental coping as these constructs had the largest direct and indirect effects on COVID-19 protection behavior. A meta-analysis examining intervention strategies based on health behavior theories concluded that perceived susceptibility and perceived severity are cues to engage in direct action behaviors (i.e., planning when, where and how to act) (Sheeran et al., 2016). Therefore, an empirically-based education and health program focusing on helping people to recognize their own ability to engage in instrumental actions may facilitate the adherence to protective measures. Promoting effective planning and thinking about specific actions that can improve the situation relates to how individuals prepare themselves (i.e., having at one's disposal hand sanitizer, handkerchiefs, and face masks) to overcome or mitigate obstacles arising from trying to comply with measures proposed by the authorities to protect individual and community health. These actions would seem especially useful for those with no direct exposure to the virus, a common situation during the onset of a pandemic. For example, the inclusion of these coping strategies in mass media dissemination messages would also enhance the effect of perceived vulnerability on the adoption of sanctioned protective measures.

Until an effective and tested vaccination rollout is completed worldwide, we will still have to live with the threat of the negative psychological, social and economic effects of COVID-19 on millions of people. Complying with scientifically sound protection measures is the most effective way of reducing the life-threatening consequences of the virus. As such, the results from this study aim toward stressing the importance of understanding how to develop effective behavioral interventions that increase a population's engagement with health measures and messages, especially when confronting unexpected and socially challenging diseases.

## Data Availability Statement

The raw data supporting the conclusions of this article will be made available by the authors, without undue reservation.

## Ethics Statement

The studies involving human participants were reviewed and approved by the Bioethics Committee of the University of Burgos (IR10/2020). The patients/participants provided their written informed consent to participate in this study.

## Author Contributions

JG-C and SU-L contributed to the conception and design of the study. JG-C, SU-L, and AP-M organized the database and performed the statistical analysis. JG-C, SU-L, AP-M, and MG-L wrote the first draft of the manuscript. All authors contributed to the manuscript revision, reading, and approving the submitted version.

## Conflict of Interest

The authors declare that the research was conducted in the absence of any commercial or financial relationships that could be construed as a potential conflict of interest.
